# Nutritional Deficiencies and Clinical Correlates in First-Episode Psychosis: A Systematic Review and Meta-analysis

**DOI:** 10.1093/schbul/sbx162

**Published:** 2017-11-30

**Authors:** Joseph Firth, Rebekah Carney, Brendon Stubbs, Scott B Teasdale, Davy Vancampfort, Philip B Ward, Michael Berk, Jerome Sarris

**Affiliations:** 1 NICM, School of Science and Health, University of Western Sydney, Sydney, Australia; 2 Division of Psychology and Mental Health, Faculty of Biology, Medicine and Health, University of Manchester, Manchester, UK; 3 Physiotherapy Department, South London and Maudsley NHS Foundation Trust, London, UK; 4 Department of Psychological Medicine, Institute of Psychiatry, Psychology and Neuroscience, King’s College London, London, UK; 5 Keeping the Body in Mind Program, South Eastern Sydney Local Health District, Sydney, Australia; 6 School of Psychiatry, University of New South Wales, Sydney, Australia; 7 KU Leuven Department of Rehabilitation Sciences, Leuven, Belgium; 8 KU Leuven Department of Neurosciences, UPC KU Leuven, Leuven, Belgium; 9 Schizophrenia Research Unit, Ingham Institute of Applied Medical Research, Liverpool, Australia; 10 Deakin University, School of Medicine, IMPACT Strategic Research Centre, Barwon Health, Geelong, Australia; 11 Florey Institute of Neuroscience and Mental Health, University of Melbourne, Melbourne, Australia; 12 Orygen, The National Centre of Excellence in Youth Mental Health and Orygen Youth Health Research Centre, Melbourne, Australia; 13 Department of Psychiatry, University of Melbourne, The Melbourne Clinic, Melbourne, Australia

**Keywords:** folic acid, cholecalciferol, nutrients, dietary, trace elements, nutrition, antioxidants

## Abstract

**Objective:**

Diet is increasingly recognized as a potentially modifiable factor influencing the onset and outcomes of psychiatric disorders. Whereas, previous research has shown long-term schizophrenia is associated with various nutritional deficiencies, this meta-analysis aimed to determine the prevalence and extent of nutritional deficits in first-episode psychosis (FEP).

**Method:**

A search of electronic databases conducted in July 2017 identified 28 eligible studies, examining blood levels of 6 vitamins and 10 minerals across 2612 individuals: 1221 individuals with FEP and 1391 control subjects. Meta-analyses compared nutrient levels in FEP to nonpsychiatric controls. Clinical correlates of nutritional status in patient samples were systematically reviewed.

**Results:**

Significantly lower blood levels of folate (*N* = 6, *n* = 827, *g* = −0.624, 95% confidence interval [CI] = −1.176 to −0.072, *P* = .027) and vitamin D (*N* = 7, *n* = 906, *g* = −1.055, 95% CI = −1.99 to −0.119, *P* = .027) were found in FEP compared to healthy controls. Synthesis of clinical correlates found both folate and vitamin D held significant inverse relationships with psychiatric symptoms in FEP. There was also limited evidence for serum level reductions of vitamin C (*N* = 2, *n* = 96, *g* = −2.207, 95% CI = −3.71 to −0.71, *P* = .004). No differences were found for other vitamins or minerals.

**Conclusions:**

Deficits in vitamin D and folate previously observed in long-term schizophrenia appear to exist from illness onset, and are associated with worse symptomology. Further research must examine the direction and nature of these relationships (ie, mediator, moderator, or marker) with clinical status in FEP. Future trials assessing efficacy of nutrient supplementation in FEP samples should consider targeting and stratifying for baseline deficiency.

## Introduction

Nutritional deficiencies, resulting from insufficient intake or absorption of nutrients critical to human health, are now a recognized risk factor for psychiatric disorders.^1,2^ For instance, excessive intake of nutritionally devoid foods is predictive of poor mental health,^3^ whereas a healthy diet reduces risk.^4^ Although previous research has focused on common mental disorders,^1,5^ recent attention has been drawn to the food intake of people with schizophrenia—who may have the worst diet, poorest metabolic health, and greatest premature mortality across all severe mental illnesses.^6–8^

Blood levels of certain nutrients are also significantly reduced in psychiatric disorders. Folate (B9) and B12 are often deficient in schizophrenia,^9,10^ and be associated with symptom severity.^11^ Furthermore, B-vitamin supplementation can significantly reduce symptoms of schizophrenia^12^ and reverse some neurological deficits associated with the disorder.^13^ This is perhaps due to the neuroprotective properties of these nutrients,^14^ or the ability of B vitamins to lower homocysteine levels, which adversely affect mental health.^14,15^ Antioxidant vitamins C and E are also reduced in long-term schizophrenia, potentially contributing to the elevated oxidative stress observed in this population.^16^

Additionally, vitamin D is implicated in schizophrenia onset, with a body of research showing developmental deficiencies in vitamin D3 increase later risk.^17–20^ Furthermore, vitamin D deficiencies persist over long-term illness and may be associated with worsened physical and mental health outcomes.^21,22^ Studies have also indicated that certain dietary minerals, such as zinc and selenium, are lowered in people with schizophrenia;^23–25^ as has been observed in other conditions such as depression.^26,27^ The direction of these nutrient/pathology associations however need to be clarified by prospective research.

Previous meta-analyses examining individual nutrient levels in individuals with long-term schizophrenia have shown clear deficits in B vitamins (folate^28^ and B12^29^), antioxidant vitamins (C and E),^16^ and vitamin D.^30^ However, which nutritional deficiencies are present at the first episode of psychosis (FEP), independent of antipsychotic treatment, has yet to be determined. This is a particularly pertinent issue, given that diet quality appears reduced from psychosis onset,^31,32^ and inflammation and oxidative stress are highest at this point of illness^16,33^; both of which may be also linked to poor nutrition. Additionally, the FEP phase has been identified as a “critical period” as firstly it is the stage where the process of neuroprogression appears most active,^34^ and secondly for reducing physical health inequalities,^35^ as the initiation of antipsychotic treatment is associated with rapid weight gain and metabolic dysfunction.^36^ Therefore, determining which nutritional deficits are present from the onset of antipsychotic treatment, and identifying which nutritional deficiencies are associated with physical and mental health outcomes in FEP, will identify key targets for dietary/nutrient interventions to improve nutritional status and attenuate neuroprogression and metabolic risk. Indeed, randomized controlled trials (RCTs) in long-term schizophrenia have already indicated that vitamin supplementation has greatest efficacy among individuals with shorter illness duration.^12^

In the current study, we used meta-analytic techniques to quantify the presence and severity of nutritional deficits in FEP, across every class of vitamin and/or dietary mineral examined in this population to date. Along with determining where deficits may exist, we also sought to systematically review the clinical correlates of nutritional status, to identify which vitamins and minerals are related to physical and mental health outcomes in FEP.

## Methods

This meta-analysis followed the PRISMA statement^37^ to ensure comprehensive and transparent reporting (supplement 1).

### Search Strategy

An electronic database search Cochrane Central Register of Controlled Trials, Health Technology Assessment Database, AMED, HMIC, MEDLINE, PsycINFO, and EMBASE was conducted on July 7, 2017. A keyword search algorithm (supplement 2) was developed to identify all studies assessing blood levels of any vitamins and/or minerals in FEP. The reference lists of retrieved articles and Google Scholar were also searched to identify any additional relevant articles.

### Screening and Selection Process

Only peer-reviewed research articles available in English were included. Eligible samples were those in which >75% of individuals were specified as “first-episode psychosis.” Studies in which the term “first-episode psychosis” was not explicitly used were only eligible if the entire patient sample was specified as either (1) currently receiving treatment from “early intervention in psychosis” services, or (2) within the first 3 years of receiving antipsychotic treatment for schizophrenia or other nonaffective psychotic disorders (as this period is broadly accepted and used as an appropriate timeframe for “early intervention” programs for psychosis).^38–40^ This criterion was applied to capture all relevant studies of patients in relatively early stages of illness and antipsychotic treatment, while excluding samples likely representative of those with more established illness. Eligible studies were those which either: (1) compared blood levels (including whole blood, plasma, serum, erythrocyte, leukocyte, or other blood components) of any vitamin and/or dietary mineral (as defined by the British Nutrition Foundation, 2016^41^) in FEP to a non-FEP control sample, or (2) reported on clinical correlates (ie, any metabolic, psychiatric, or neurocognitive parameters assessed using a clinically validated measure) of vitamin/mineral levels in FEP samples. For inclusion in the systematic review, no restriction was placed on the nature of control sample used. However, meta-analyses used only the data from studies which compared FEP to “healthy” control samples (ie, individuals with no psychiatric diagnosis).

### Data Extraction

Articles were screened for eligibility by 2 independent reviewers (J.F. and R.C.). Disagreements were resolved through discussion until consensus was reached. Where further information or study data were required, the corresponding authors of the respective articles were contacted twice over the period of 1 month to request this. This was attempted for one paper but data were not retrieved.^42^ A systematic tool was used to extract the following data from each study:

1. *Study characteristics:* study design, country, sample size (*n*).
2. *FEP sample:* age, % male, inpatient/outpatient status, duration of untreated psychosis, medication type, and duration of antipsychotic treatment.
3. *Control sample:* age, % male, clinical/healthy population, any sociodemographic characteristics matched to FEP sample for.
4. *Study findings:* blood levels of vitamin/minerals in FEP and controls, outcomes of all reported statistical comparisons between groups, relationships with any physical/psychiatric/neurocognitive assessment measures.

### Statistical Analyses

Meta-analyses were performed in Comprehensive Meta-Analysis 2.0.^43^ A random effects model was applied to all analyses to account for methodological heterogeneity between eligible studies,^44^ in terms of differences in both blood sampling procedures and assay measures used across studies. Individual analyses were performed for each vitamin and mineral examined in FEP patients, using pooled comparisons with blood levels observed in nonpsychiatric control samples. Where studies used multiple measures of an individual vitamin or mineral, the more complex measure which best reflects bioavailable levels or long-standing nutrient was preferentially used. The overall difference between FEP and control groups for each vitamin/mineral was computed as Hedge’s G from the raw (ie, unadjusted) mean levels reported in each study.

Variance between studies was assessed using Cochran’s *Q* and *I*2 values, both of which estimate the degree of variance resulting from between-study heterogeneity, rather than chance. For all statistically significant findings, Egger’s regression test was applied to quantify the risk of publication bias. Additionally, a “Fail-Safe N”^45^ was calculated to determine the number of unpublished null studies which would invalidate the findings, and Duval and Tweedie’s trim-and-fill analysis was applied. We also performed post hoc sensitivity analyses to assess if comparable differences were still observed when excluding outlier studies with very high effect sizes.

Finally, for all individual study findings which could not be combined in meta-analyses, we produced a systematic narrative synthesis reporting on: (1) differences between FEP samples and other “non healthy” (ie, clinical/psychiatric) populations, and (2) all clinical correlates of vitamin/mineral levels in FEP.

## Results

**Fig. 1.**
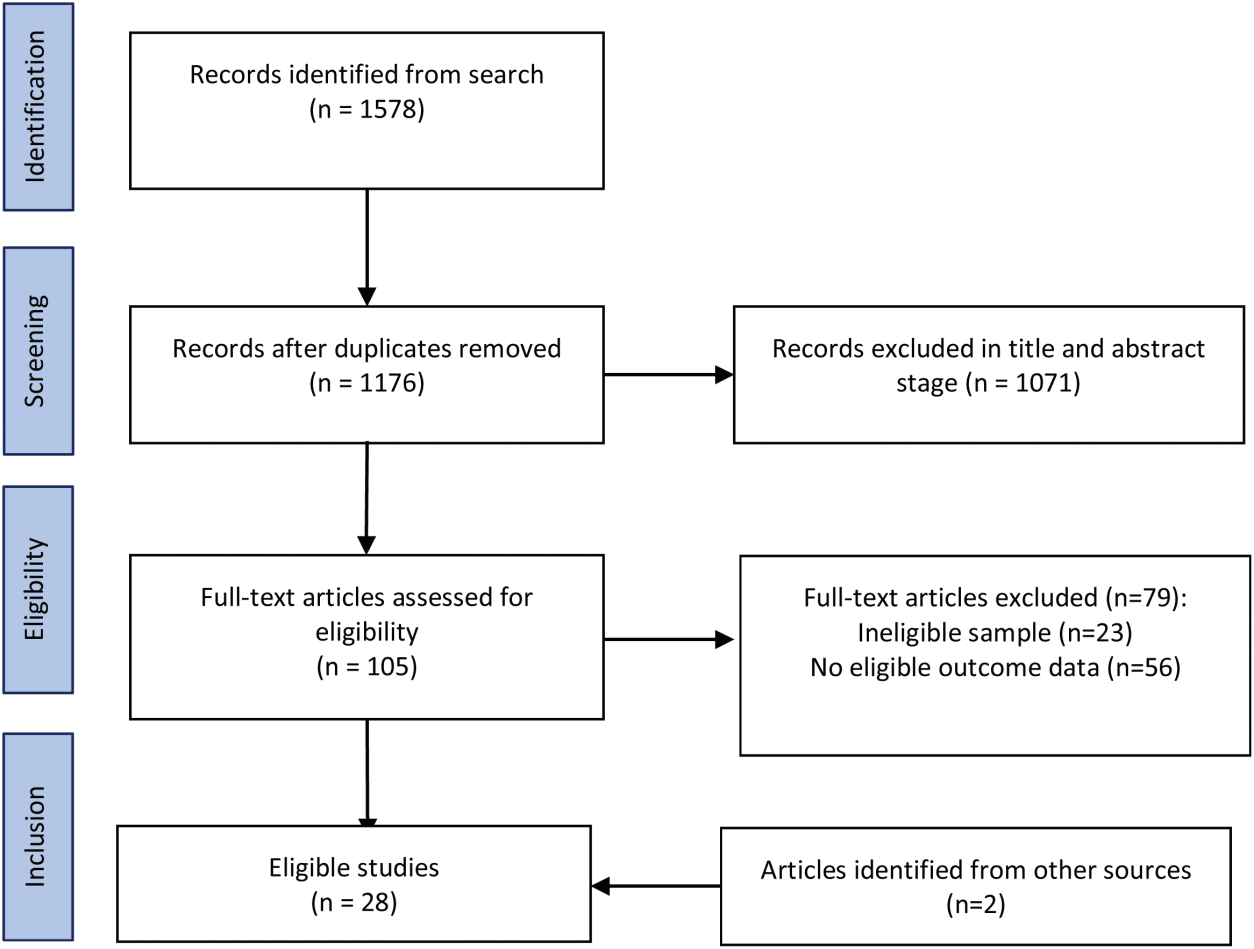
PRISMA search diagram.

The initial database search was performed on July 7, 2017. The search returned 1578 results, reduced to 1176 after duplicates were removed. A further 1071 articles were excluded after and abstract screening. Full text versions were retrieved for 105 articles, of which were 26 eligible for inclusion. A further 2 eligible articles were identified from an additional search of Google Scholar. Full details of the search results and reasons for exclusion and are summarized in figure 1.

A total of 28 articles, reporting data from 24 unique samples of 2612 participants (1221 FEP, 1391 controls) were included. These assessed differences in blood levels of 6 vitamins (A, B12, C, D, E, and folate) and 10 minerals (zinc, magnesium, sodium, potassium, calcium, copper, chromium, iron, manganese, and selenium). Four studies were conducted in India, 3 in Pakistan and China, 2 in United States of America, United Kingdom, Turkey, Spain, and Nigeria, and individual studies in Poland, Romania, Norway, and Singapore. Full details and findings of all included studies are shown in table 1a–d.

**Table 1.** Study Details

Study (Country)	Population Studied + Treatment Information	FEP Sample Age (Yrs) % Male	Ctrl Sample Age (Yrs) % Male	DUP (Yrs)	Study Design and Comparator	Vitamin/Minerals Measured	Differences Found	Clinical Correlates
*a. B vitamin studies*								
Ayesa-Arriola et al.^90^ (Spain)	In + outpatients	*N* = 139	*N* = 99	1.13	Cross-sectional with healthy controls	Vitamin B12 (serum)	Serum folate and vitamin B12 did not differ between groups	None examined
	Medication-naïve	32.9 yrs	27.0 yrs			Folate (serum)		
	FEP classification	54% male	59% male					
Ipcioglu et al^46^ (Turkey)	Inpatients	*N* = 14	*N* = 34	*N* = 30	Cross-sectional with healthy and psychiatric controls	Vitamin B12 (serum)	Serum folate and vitamin B12 did not differ between groups	None examined
	Medication n/s	21.8 yrs	30.6 yrs	33 yrs		Folate (serum)		
	FEP classification	50% male	50% male [HC]	53% male (Depression)				
Kale et al.^52^ (India)	Outpatients	*N* = 31	*N* = 48	0.52	Cross-sectional with healthy controls	Vitamin B12 (plasma)	Significantly lower plasma folate (*P* = .02) and RBC (*P* = .01), and lower B12 plasma (*P* = .09)	Vitamin B12 showed trend level negative correlation with positive symptoms on PANSS, but no significant correlations for plasma folate and PANSS scores
	Medication-naïve	33 yrs	34 yrs			Folate (plasma + RBC)		
	FEP classification	56% male	55% male					
Misiak et al.^51^ (Poland)	67% SGA	*N* = 135	*N* = 146	1.16 yrs	Cross-sectional compared with healthy controls	Vitamin B12 (serum)	Significantly lower serum folate in FEP group (*P* < .001) but no difference in B12	Inverse correlation between PANSS negative scores and serum B12, but no relationship found for folate or other clinical correlates
	18% Medication-naïve	27.2 yrs56% male	27.6 yrs46% male			Folate (serum)		
	15% FGA							
	FEP classification							
Misiak et al.^50^ (Poland)	Subsample of above	*N* = 56	*N* = 53	Not specified	Cross-sectional compared with healthy controls	Vitamin B12 (plasma)	Significantly lower plasma folate in FEP group (*P* < .001) but no difference in B12	Plasma B12 correlated inversely with severity of negative symptoms. Higher folate levels were associated with lower general symptom scores.
		27.2 yrs	25.7 yrs			Folate (plasma)		
		56% male	49% male					
Misiak et al.^54^ (Poland)	Subsample of above	*N* = 39	N/A	Not specified	12 week observational study of SGAs	Vitamin B12 (serum)	N/A	12 weeks of treatment with olanzapine, but not risperidone, was associated with significant reductions in folate and B12
		26.0 yrs				Folate (serum)		
		60% male						
Misiak et al.^91^ (Poland)	Subsample of above	*N* = 83	N/A	Not specified	Cross-sectional	Vitamin B12 (serum)Folate (serum)	N/A	Folate or B12 plasma were not associated with childhood trauma
		25.1 yrs (m)						
		28.8 yrs (f)						
		57% male						
Song et al.^48^ (China)	Inpatient	*N* = 46	*N* = 30	0.65 yrs	Cross-sectional compared with healthy controls	Folate (serum)	Serum folate did not differ between groups	Inverse relationship between serum levels of folate and PANSS total and PANSS negative symptom scores
	Medication-naïve	22.5 yrs	24.3 yrs					
	FEP classification	61% male	57% male					
Xuimei et al.^49^ (China)	Inpatient	*N* = 60	*N* = 60	Not specified	Cross-sectional compared with healthy controls	Folate (serum)	Serum folate significantly lower in the FEP group (*P* < .01).	Negative correlation between serum levels of folate and negative symptoms (PANSS) but not positive symptoms, general psychopathology or cognitive function scores
	Medication-naïve	22 yrs	23 yrs					
	FEP classification	57% male	53% male					
***b. Vitamin D studies***								
Crews et al.^92^ (UK)	Inpatient	*N* = 69	*N* = 69		Cross-sectional with healthy controls	Vitamin D (serum)	Serum level vitamin D significantly lower in FEP group than controls (*P* < .001) and higher level of deficiencies (OR = 2.99, *P* = .008)	No correlation between vitamin D and time as an inpatient. No difference according to antipsychotic or anticonvulsant use but vitamin D levels higher in SSRI users than nonusers (*P* = .04).
	86% antipsychotic (not specified)	31 yrs39% male	31 yrs39% male					
	25% SSRIs							
	6% anticonvulsant FEP							
Graham et al.^57^ (USA)	Outpatient	*N* = 20	*N* = 20	<5 yrs	Cross-sectional with healthy controls	Vitamin D (serum)	No significant difference in vitamin D serum levels	More severe negative and total symptoms (PANSS) associated with lower vitamin D levels (*P* = .03). Trend level association found for positive symptoms. No relationship with depressive symptoms (CDRS). Lower vitamin D associated with more severe cognitive deficits including verbal fluency scores in patient group.
	Medicated <16 weeks FEP	23 yrs60% male	25 yrs60% male					

Malik et al.^54^ (Pakistan)	Inpatients	*N* = 100	*N* = 100	Not specified	Cross-sectional with general population	Vitamin D (serum)	Vitamin D levels significantly lower in people with newly diagnosed schizophrenia compared with controls	None reported
	Unmedicated “newly diagnosed”	Age and gender not reported	Age and gender not reported					
Nerhus et al.^55^	In + outpatient	*N* = 71	*N* = 142	2.8 yrs	Cross-sectional with healthy controls and established SZ	Vitamin D (serum)	No significant differences in vitamin D with healthy controls	Lower levels of vitamin D related to higher levels of depressive symptoms in FEP. No significant relationship between vitamin D and positive or negative symptoms (PANSS).
	83% medicated	27 yrs	28 yrs					
	77% AP; 3% lithium; 6% antiepileptic; 24% AD; 6% hypnotic	65% male	65% male[HC]					
	FEP classification							
			*N* = 71				No significant differences in vitamin D of people experiencing a FEP and those with established SZ	
			28 yrs					
			65% male					
			[Established SZ]					
Salavert et al.^56^ (Spain)	Medication-naïve FEP	*N* = 45	*N* = 22	Not specified	Cross-sectional with healthy controls	Vitamin D (serum)	Significantly reduced vitamin D levels in FEP sample compared to control condition	Patients who later received diagnosis of schizophrenia had trend-level lower vitamin D than those who were diagnosed with other psychoses (*P* = .06)
		33.7 yrs	36.1 yrs					
		60% male	27.3% male					
Yee et al.^58^ (Singapore)	<4 weeks AP FEP classification	*N* = 3129 yrs48% male	*N* = 3129 yrs45% male	1.8 yrs	Cross-sectional with healthy controls	Vitamin D (serum and bioavailable levels)	No significant difference in serum levels of vitamin D between groups, but controls had significantly higher levels of serum bioavailable vitamin D (*P* = .05)	Significant negative association between vitamin D and negative symptoms (PANSS) even after controlling for gender, ethnicity, and DUP
						Calcium (serum)		

Zhu et al.^93^ (China)	Outpatients	*N* = 93	*N* = 93	<5 yrs	Cross-sectional with family members	Vitamin D (plasma)	Plasma vitamin D significantly lower in FEP than healthy family members (*P* < .001). Mean vitamin D levels 40% lower in patients. People in lower quartiles of vitamin D levels had significantly increased proportions of SZ.	None reported
	<16 weeks AP	30 yrs	43 yrs					
	FEP classification	41% male	52% male					
***c. Antioxidant vitamin studies***								
Dadheech et al.^59^ (India)	<40 years old“Newly diagnosed”	*N* = 3072% male	*N* = 40	Not specified	Cross-sectional with healthy controls and older SZ	Vitamin E (plasma)	Patient sample had significantly lower vitamin E and C. Older patients had lower vitamin E than younger patients.	Not reported
			73% male [HC]			Vitamin C (plasma + leukocyte)		
			*N* = 28					
			>40 years old					
			72% male [older SZ]					
Dakhale et al.^62^ (India)	Inpatients	*N* = 40		Not specified	RCT of vitamin C	Vitamin C (plasma)	N/A	Negative symptoms (BPRS) were significantly reduced after 8 weeks of treatment with 500 mg/day of vitamin C compared with placebo (*P* < .01). Significant and negative correlation was found between plasma ascorbic acid levels and BPRS score (*r* = −0.38, *P* < .05).
	Unmedicated	38.5 yrs						
Sarandol et al.^94^ (Turkey)	Medication naïve	*N* = 26	*N* = 25	Not specified	Longitudinal single arm trial + cross-sectional with healthy controls	Vitamin E (plasma)	No significant difference in plasma levels of vitamin E	None reported
	FEP classification	26 yrs	24 yrs					
		39% male	40% male					
Scottish Schizophrenia Research Group^61^ (UK)	Inpatients	*N* = 30	*N* = 30	Not specified	Cross-sectional with general population	Vitamin E (serum)	Serum levels of vitamin E significantly lower in FEP group, but no difference for vitamin A. 77% of FEP and 70% controls had ratio of vitamin E to cholesterol <5 (level necessary to protect against heart disease).	Vitamin levels not related to psychiatric symptoms (PANSS, CGI).
	Medication-naïveFEP classification	28 yrs (m); 33 yrs (f)	30 yrs70% male			Vitamin A (serum)		
		70% male						
Surapaneni^60^ (India)	“Newly diagnosed”	*N* = 48	*N* = 48	Not specified	Cross-sectional with healthy controls	Vitamin E (plasma)	Significantly lower vitamin E and vitamin C in patient sample	None reported
		63% male	63% male			Vitamin C (plasma)		
***d. Dietary mineral studies***								
Akinladel et al.^63^ (Nigeria)	<3 years illness	*N* = 19	*N* = 30 [HC]		Cross-sectional compared with healthy controls and long term SZ	Sodium, potassium (serum)	Significantly lower potassium and sodium in FEP than controls. No difference between FEP and long- term SZ.	None reported
	Medication naïveFEP classification		*N* = 41 [Established SZ]					

Arinola and Idonije ^95^ (Nigeria) + Arinola et al.^65^ (Nigeria)	InpatientMedication-naive	*N* = 1524 yrs73% male	*N* = 20	0.15 yrs	Cross-sectional compared with healthy controls and medicated SZ	Zinc, iron, manganese, chromium, selenium, magnesium, copper (all plasma)	Iron, selenium, and chromium were all significantly higher in unmedicated FEP patients compared with healthy controls (*P* < .01). No significant difference was found for other minerals.	Zinc was significantly lower and manganese and chromium were significantly higher in unmedicated than medicated SZ
			28 yrs60% male[HC]					

			*N* = 20					
			27 yrs					
			65% male [Established SZ]					
Gunduz-Bruce et al.^96^ (USA)	<6 months medication	*N* = 16 25.7 yrs75% male*N* = 75Age and gender not reported	*N* = 28 28.3 yrs36% male*N* = 25Age and gender not reported	Not specified	Cross-sectional compared with healthy controls	Sodium (plasma)	Plasma sodium levels significantly higher in patient group (*P* = .05)	None reported
	FEP classification							

Jamil et al.^98^ (Pakistan)	Inpatient			Not specified	Cross-sectional compared with healthy controls.	Calcium (serum)	Higher levels of serum calcium (*P* = .04) and sodium (*P* = .01), and lower levels of potassium (*P* < .01) and magnesium (*P* = .06) were observed in newly diagnosed patients compared with controls	None reported
	Unmedicated“Newly diagnosed”					Sodium (serum)Potassium (serum)Magnesium (serum)		
	
		
Nawaz et al.^97^ (Pakistan)	Outpatients	*N* = 12	*N* = 19	Not specified	Cross-sectional compared with siblings and established SZ	Chromium, zinc, copper, magnesium, iron, manganese, selenium (all plasma)	No significant difference in any trace metals in the newly diagnosed group compared with their siblings	None reported
	Unmedicated“Newly diagnosed”	Age and gender not reported	Age and gender not reported[Sibling]					

			*N* = 22				No significant difference between newly diagnosed and established SZ	
			Age and gender not reported [Established SZ]					
Nechifor et al.^66^ (Romania)	Inpatient	*N* = 56	*N* = 20	Not specified	Longitudinal; 3 week trial of haloperidol v risperidone compared with controls	Magnesium (plasma + erythrocyte) calcium, zinc, copper	Significantly lower erythrocyte magnesium and zinc levels in FEP group (*P* < .01) but no difference in plasma magnesium, calcium or copper	Both haloperidol and risperidone treatments were associated with increased levels of erythrocyte
	71% SGA29% FGA“Newly treated”	38 yrs (median)43% male	Not reported					
								Magnesium and plasma zinc after 3 weeks
	

*Note:* AP, antipsychotic; DEP, depression; DUP, duration untreated psychosis; FEP, first-episode psychosis; FGA, first generation antipsychotics; N/A, not applicable; PANSS, Positive and Negative Syndrome Scale; RBC, red blood count; RCT, randomized controlled trial; SANS, Scale for the assessment of negative symptoms; SAPS, Scale for the assessment of positive symptoms; SGA, second generation antipsychotics; SSRI, selective serotonin reuptake inhibitors; SZ, schizophrenia; yrs, years.

### B Vitamins in FEP

Nine studies, with 6 independent samples, reported on B vitamin levels across 872 participants: 425 with FEP, all of whom were medication-naïve (except for one study where this was no specified; table 1), and 447 controls. Two B vitamins were examined: folate (B9) and cobalamin (B12).

**Fig. 2.**
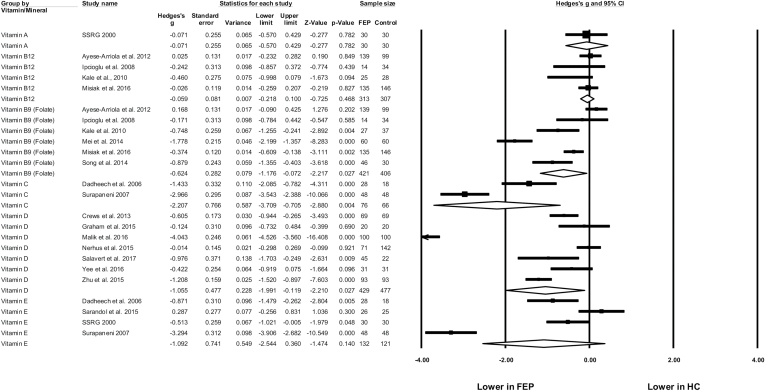
Meta-analysis of blood levels of vitamins in first-episode psychosis (FEP) and healthy controls (HC). Box size represents study weighting. Diamond represents overall effect size and 95% confidence intervals.

Random effects meta-analyses found significantly lower blood levels of folate in FEP compared with healthy controls (figure 2), with a moderately large effect size (*N* = 6, *n* = 827, *g* = −0.624, 95% CI = −1.176 to −0.072, *P* = .027). There was significant heterogeneity (*Q* = 66.0, *P* < .01, *I*^2^ = 92.4%), and a range of blood measures applied across studies, showing significant differences between FEP and controls in plasma, serum, and red blood cell levels (table 1). There was no evidence of publication bias (Eggers regression, *P* = .16) and the trim-and-fill analysis did not identify any outlier studies. Furthermore, the fail-safe N was 71, indicating 71 additional null studies would be required to make the observed difference nonsignificant.

Four studies (*n* = 620) examined blood levels of vitamin B12, finding no significant difference between FEP and healthy controls (*g* = −0.059, 95% CI = −0.22 to 0.10, *P* = .468, *Q* = 2.96, *I*^2^ = 0%). A single study compared serum folate and vitamin B12 levels in FEP to people with depression,^46^ finding no significant differences between the 2 patient groups. However, no firm conclusions could this be drawn due to the low number of FEP patients examined (*n* = 14).

Table 1a shows all clinical correlates of B vitamin levels in FEP. Five studies examined relationships between folate levels and psychiatric symptoms (measured using the Positive and Negative Syndrome Scale; “PANSS”^47^).^48–52^ Only one found a significant correlation with PANSS total scores.^48^ However, 3 found significant correlations between serum folate and PANSS subscales; with lower folate levels predicting more severe negative^48,49^ or general^53^ symptoms. No associations were found for positive symptoms or cognition.

Three studies examined clinical correlates of vitamin B12 in FEP. One found trend-level correlation with reduced positive symptoms,^52^ and 2 found significant inverse correlation between B12 and negative symptom scores.^51,53^ One further study examining the impact of antipsychotic medications found significant reductions in serum levels of both folate and B12 after 12 weeks of treatment with olanzapine, but no reduction from risperidone in FEP.^50^ Finally, one study found that neither folate or B12 levels were related to childhood trauma.^53^

### Vitamin D in FEP

Seven studies examined blood levels of vitamin D using plasma and serum measures across 906 participants; 429 with FEP, 477 controls (table 1b). Meta-analyses comparing FEP to healthy control samples (all matched for age and ethnicity) showed that people with FEP had reduced vitamin D levels; with a large, significant difference between the 2 groups (*g* = −1.055, 95% CI = −1.99 to −0.119, *P* = .027) (figure 2). There was significant heterogeneity but no evidence of publication bias (*Q* = 216.6, *P* < .01, *I*^2^ = 97.2%, Egger’s regression *P* = .27, Fail Safe *N* = 265). A sensitivity analysis excluding the single study with large effect size (*g* = −4.04) which did not use established FEP criterion (classifying patients as “newly diagnosed”)^54^ found the difference was still significant among 6 remaining studies (*g* = −0.554, 95% CI = −1.00 to −0.113, *P* = .014). A study comparing vitamin D in FEP and multi-episode schizophrenia found no significant differences between groups.^55^ However, one study found that the FEP patients later diagnosed with schizophrenia had trend-level lower vitamin D than FEP patients who were later diagnosed with other psychoses (*P* = .06).^56^

Three studies examined correlations between vitamin D and psychiatric symptoms; all finding some link between low vitamin D and worse mental health (table 1b). Graham et al^57^ found serum levels of vitamin D were negatively associated with PANSS totals and negative subscale scores, overall neurocognitive functioning, and specific tests of verbal fluency. There was also a trend-level association (*P* = .054) with PANSS positive symptoms. Yee et al^58^ found low vitamin D levels were only associated with greater PANSS negative symptoms, whereas Nerhus et al^55^ only observed a significant relationship with depressive symptoms (PANSS factor scale).

### Antioxidant Vitamins in FEP

Five studies assessed blood levels of antioxidant vitamins (A, C, and E) in FEP (table 1c). As shown in figure 2, significant differences were only observed for vitamin C (*g* = −2.207, 95% CI = −3.71 to −0.71, *P* = .004) from 2 studies observing large deficits of vitamin C in FEP samples.^59,60^ However, there was a small sample (*n* = 96), substantial heterogeneity (*Q* = 11.9, *P* < .01, *I*^2^ = 91.6%), and studies did not report the specifics of FEP classification (samples only described as “newly diagnosed for schizophrenia”). Differences in vitamin E were nonsignificant (*N* = 4, *n* = 253, *g* = −1.09, 95% CI = −2.54 to 0.36, *P* = .14). The sole study examining vitamin A levels in FEP (*n* = 30) found no difference from healthy controls (*n* = 30).^61^

Regarding clinical correlates of antioxidant vitamins, neither A or E levels correlated with psychiatric symptoms (measured with PANSS or Clinical Global Impression scales).^61^ Nonetheless, Dakhale et al^62^ found that higher plasma vitamin C in newly diagnosed schizophrenia patients receiving vitamin C supplementation (*n* = 40) were associated with greater symptomatic improvement over 8 weeks (assessed with the Brief Psychiatric Rating Scale) (*r* = −.38, *P* < .05).

### Dietary Minerals in FEP

**Fig. 3.**
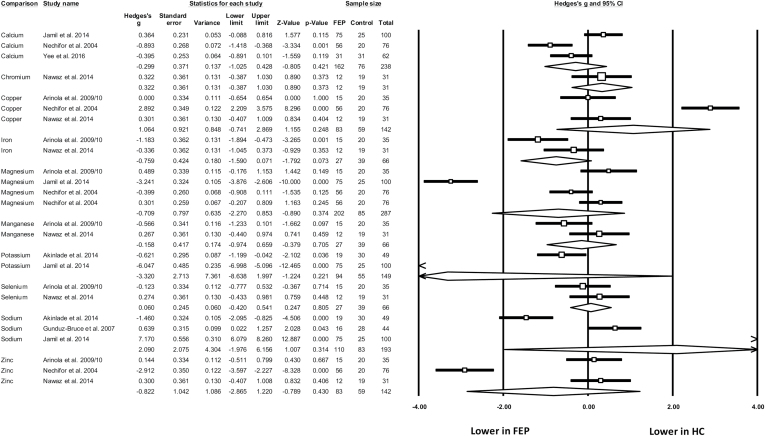
Meta-analysis of blood levels of dietary minerals in first-episode psychosis (FEP) and healthy controls (HC). Box size represents study weighting. Diamond represents overall effect size and 95% confidence intervals.

Ten dietary minerals were assessed across 8 studies (table 1d) with 480 participants (224 FEP, 173 healthy controls, 83 older/longer-term schizophrenia). Only one study used the term “first-episode psychosis,”^58^ with all others describing their samples as “medication-naïve” or “newly treated/diagnosed schizophrenia patients.” There were 3 studies examining blood levels of calcium, copper, magnesium, sodium, and zinc; 2 studies for iron, manganese, potassium, and selenium; and 1 study for chromium. Meta-analyses found no significant differences between FEP samples and healthy controls for any dietary mineral (figure 3).

When comparing patient groups, 2 studies found no difference in mineral levels between newly diagnosed patients and those with established schizophrenia.^63,64^ However, Arinola et al^65^ found that zinc, manganese, and chromium were all significantly lower in newly diagnosed, medication-naïve patients (*n* = 15) compared to medicated schizophrenia patients (*n* = 20). Furthermore, in a longitudinal study, Nechifor et al^66^ found that both magnesium and zinc were raised significantly following antipsychotic treatment (with either haloperidol or risperidone). No studies examined correlations between mineral levels and symptomology in FEP.

## Discussion

This is the first study to examine serum nutrient status in FEP, and the first to show that compared to nonpsychiatric controls, reduced nutritional status exists independently, and in some cases, before antipsychotic treatment. To date, meta-analyses have only examined nutritional status in people with long-term schizophrenia,^28–30^ and each of these have focused on single nutrients in isolation.^28–30^ In this study, we included all vitamins and minerals examined in FEP to date, to identify which particular nutrient levels may act as specific biomarkers and/or therapeutic targets for this population. Our systematic search found 28 studies with 24 independent samples comparing blood levels of 6 vitamins and 10 dietary minerals in 1221 people with FEP to 1391 controls. Random effects meta-analysis found significant reductions in folate, vitamin D, and vitamin C among people with FEP compared to nonpsychiatric controls, with no significant differences for other vitamins or minerals.

The strongest evidence was found for vitamin D deficits; with pooled data from 7 independent studies showing a large, significant reduction across 429 individuals with FEP compared to 477 nonpsychiatric controls (*g* = −1.1, *P* = .027), all matched for age and ethnicity. Given the prevalence of vitamin D deficiencies in long-term schizophrenia,^30^ and the compelling evidence for low vitamin D status during brain development being linked to schizophrenia onset,^67^ it is perhaps unsurprising that FEP is associated with lower vitamin D. However, the extent of the deficit in FEP samples compared to the general population observed in this meta-analysis is troubling, especially considering that vitamin D levels are often low even among healthy adults,^68^ with 20%–40% of young adults in UK population showing insufficiencies.^69^ Furthermore, within the FEP samples, lower vitamin D levels were found to be associated with more severe symptomology.^55,57,58^

Currently, there is an absence of RCTs examining the efficacy of vitamin D supplementation as an adjunctive treatment in FEP—although previous studies have shown vitamin D supplements used during childhood can reduce the risk of developing schizophrenia.^20^ The efficacy of vitamin D supplementation in young people with FEP is currently being examined in a placebo-controlled RCT, the “D-Fend” study (“Vitamin D First Episode Neuroprotection Design”) (ISRCTN12424842). This will provide the first insights into potential benefits for this population. Vitamin D is an essential nutrient for both metabolic and neurological health.^70,71^ Thus, supplementing at the first-episode phase may attenuate the metabolic and neurological abnormalities which arise during the early stages of treatment. Indeed, preliminary research has already shown that vitamin D levels hold positive correlations with metabolic health,^21^ along with brain structure and functioning in people with psychosis.^57,72^

We also found that FEP samples had significantly lower serum folate than nonpsychiatric controls; as has previously been observed for long-term schizophrenia.^28^ Folate deficiencies, even in the general population, are among the most widespread of all nutritional deficiencies globally, and adversely affect intellectual development and mortality risk.^73^ Folate also plays an important role in maintaining neuronal integrity^74^ and lowering levels of homocysteine—which has been linked to aetiology of schizophrenia.^15^ Indeed, 2 studies also found higher serum folate correlated with reduced negative symptoms in FEP,^48,49^ as has been previously observed in long-term schizophrenia.^11,13^

Interestingly, the one study which measured dietary folate intake found that reduced folate levels in FEP could not be accounted for by differences in diet.^52^ Instead, genotypic differences in folate absorption efficiency may be responsible for deficiencies observed in schizophrenia.^15,52,75^ In long-term schizophrenia, this hypothesis is supported by strong experimental evidence showing that symptomatic benefits from standard folic acid supplements are moderated by genotypic differences in absorption,^75,76^ whereas administration of “L-methlyfolate” (the most bioactive folate type; readily absorbed regardless of genotype) significantly reduces negative symptoms across entire schizophrenia samples.^13^ Symptomatic benefits of L-methylfolate have also been shown in multiple RCTs in people with depression.^77,75^ Although there are no RCTs of L-methylfolate to date in individuals with FEP, research in long-term schizophrenia samples suggests that patients with shorter illness durations are more responsive to B vitamin treatments.^12^ Given the very low side-effect profile of L-methylfolate (with RCTs in other populations administering over 100 times the recommended daily requirement without any negative effects),^78^ this could potentially be trialed as an adjunctive treatment in FEP, or even as a prevention strategy for individuals identified as at-risk of psychosis. Additionally, B12 (another homocysteine-reducing B vitamin) may also be as useful adjunctive treatment, as although no pooled differences were observed between FEP and controls for this nutrient, studies consistently indicated that higher B12 was related to better mental health in FEP.^51–53^

Although only examined in 2 studies, vitamin C was also significantly reduced in FEP. This is concordant with data suggesting low fruit and vegetable intake in this population.^79^ Additionally, a single RCT in patients undergoing first antipsychotic treatment has shown 500 mg of vitamin C per day significantly reduces psychiatric symptoms.^62^ Furthermore, increases in plasma vitamin C were correlated with symptomatic improvements following the trial. Meta-analyses of other antioxidant vitamins A and E found no evidence for deficits in FEP.

However, a limitation of this meta-analysis is that despite the large number of total studies, certain individual nutrients were only examined in a small number of FEP samples—reducing statistical power to detect significant differences. Therefore, although the review provides evidence that folate and vitamin D are reduced in FEP compared to healthy populations, the nonsignificance of differences for other vitamins does not necessarily rule these out as deficient. Indeed, further adequately powered studies could ultimately find a spectrum of deficiencies impacting on the physical and mental health of people with FEP. Additionally, the lack of significant differences found between FEP samples and other psychiatric populations, such as depression^46^ and long-term schizophrenia,^55,63,64^ could be due to limited sample sizes in respective studies. Thus, further research is required to determine if any nutritional deficiencies are particularly pronounced in FEP, or if these deficiencies are prevalent across all phases/types of severe mental illness.

Another limitation is that the causative and mechanistic links between nutritional deficiencies and mental health outcomes has yet to be established.^80^ Further longitudinal and interventional research in individuals identified at “ultra-high risk” for psychosis would provide valuable insights into both the predictive value of nutritional deficiencies in the onset of psychosis, along with potentially determining if nutritional supplementation can confer any benefit for reducing psychosis risk. A note of caution is warranted, as in disorders such as cardiovascular disorders and cancer, where nutrient deficiencies have also been noted, the research has been underwhelming in supporting supplementation. For instance, while vitamin D insufficiency is widespread across noncommunicable medical conditions, RCTs of vitamin D supplementation in cardiovascular disorders, cancer, and osteoporosis have generally failed to robustly support causative hypotheses.^80–82^ It should also be considered that although nutritional deficiencies could feasibly exacerbate psychiatric symptoms, it is also possible that therapeutic benefits of supplementation may not be reliant upon vitamin/mineral deficiencies. For instance, beneficial effects of other nutrient-based adjunctive treatments (such as NAC^83^ and Taurine^84^) are not due to restoring specific nutritional deficits, but instead attributed to these amino acids targeting pathological neurological processes.^83,84^ Therefore, even in the absence of clear deficiencies, certain nutrients may confer positive effects in FEP through the neurochemical properties of these compounds, regardless of deficiency status.

Given the marked reductions in vitamin D and folate observed in FEP compared to nonpsychiatric samples, future research should also examine what proportion of individuals with FEP reach clinical thresholds for nutritional deficiencies in comparison to established reference values, which in turn could have implications for routine screening of nutritional deficiencies in FEP. Additionally, much of the emphasis for dietary intervention in schizophrenia so far lies with reducing over-consumption of obesogenic foods.^7,85^ Thus, this review also holds implications for dietary interventions to further emphasize the importance of consuming a good quality diet containing adequate nutrients. Indeed, large-scale epidemiological studies show that low levels of both vitamin D and folate are linked to cardiovascular mortality.^86,87^ Further efforts toward resolving these deficiencies holds promise to reduce the physical health inequalities observed in schizophrenia. Additionally, multi-ingredient “nutraceuticals,” which combine various beneficial nutrients to target specific nutritional deficits and neurological processes implicated in psychiatric disorders,^88,89^ may also provide safe and effective adjunctive treatments for FEP. These data also support stratification of individuals based on nutrient intake/status as criteria for nutraceutical interventions and in trial design. Given the clear lack of experimental evidence in this area,^12^ there is a now a clear need for clinical trials to evaluate the use of whole diet interventions as well as nutrient-based interventions for improving the typically poor recovery rates observed among people with FEP.

## Supplementary Material

Supplementary data are available at *Schizophrenia Bulletin* online.

## Funding

J.F. is supported by a Blackmores Institute Fellowship and an MRC Doctoral Training Grant P117413F07. J.S. is supported by an NHMRC Research Fellowship (APP1125000) and has received either presentation honoraria, travel support, clinical trial grants, book royalties, or independent consultancy payments from Integria Healthcare & MediHerb, Pfizer, Scius Health, Key Pharmaceuticals, Taki Mai, Bioceuticals & Blackmores, Soho-Flordis, Healthworld, HealthEd, HealthMasters, Elsevier, Chaminade University, International Society for Affective Disorders, Complementary Medicines Australia, Terry White Chemists, ANS, Society for Medicinal Plant and Natural Product Research, UBiome, Omega-3 Centre, the National Health and Medical Research Council, CR Roper Fellowship. M.B. is supported by a NHMRC Senior Principal Research Fellowship (GNT1059660) and has received Grant/Research Support from the NIH, Cooperative Research Centre, Simons Autism Foundation, Cancer Council of Victoria, Stanley Medical Research Foundation, MBF, NHMRC, Beyond Blue, Rotary Health, Geelong Medical Research Foundation, Bristol Myers Squibb, Eli Lilly, Glaxo SmithKline, Meat and Livestock Board, Organon, Novartis, Mayne Pharma, Servier, Woolworths, Avant, and the Harry Windsor Foundation, has been a speaker for Astra Zeneca, Bristol Myers Squibb, Eli Lilly, Glaxo SmithKline, Janssen Cilag, Lundbeck, Merck, Pfizer, Sanofi Synthelabo, Servier, Solvay, and Wyeth, and served as a consultant to Allergan, Astra Zeneca, Bioadvantex, Bionomics, Collaborative Medicinal Development, Eli Lilly, Grunbiotics, Glaxo SmithKline, Janssen Cilag, LivaNova, Lundbeck, Merck, Mylan, Otsuka, Pfizer, and Servier. R.C. is funded by an Economic and Social Research Council grant (ESJ5000991). S.T. is funded by the South Eastern Sydney Local Health District. B.S. is part funded by the National Institute for Health Research (NIHR) Biomedical Research Centre at South London and Maudsley NHS Foundation Trust and King’s College London. The views expressed are those of the author(s) and not necessarily those of the NHS, the NIHR or the Department of Health.

## Supplementary Material

Supplement 1

Supplement 2
