# Supplementary material for: Nutritional Deficiencies and Clinical Correlates in First-Episode Psychosis: A Systematic Review and Meta-analysis
Source: Schizophr Bull. 2017 Nov 30;44(6):1275–92. doi: 10.1093/schbul/sbx162 (PMC6192507; doi:10.1093/schbul/sbx162)
Supplement: Supplement 2 [file sbx162_suppl_supplement_2.doc]

| ***Category*** | **Search words used (‘OR’ terms)** |
| --- | --- |
| *Population* | Schizophr*; Schizoaffective; Psychosis; Psychotic; Psychoses; Antipsychotic*  AND  first episode; first onset; recent onset; early onset; first admission; early intervention; young; youth; early psychosis; adolescen*; medication free; unmedicated; naïve; never medicated; first treat*; newly diagnosed; recently diagnosed. |
| *Vitamins* | Vitamin B*; Thiamine; Riboflavin; Niacin; Niacinamide; Nicotinic Acid; Pantothenic; Pyridox*; Biotin; *folate; Folinic Acid; Folic acid; Cyancobalamin; Methylcobalamin; Cobalamin; B12; homocysteine; vitamin A; retinol; beta carotene; vitamin E; tocopherol; alpha-tocopherol; vitamin D; vitamin D2; vitamin D3; Cholecalciferol; vitamin C; ascorbic acid; vitamin K; phylloquinone |
| *Minerals* | Mineral*; trace element*; trace metal*; heavy metal*; potassium; sodium; calcium; copper; magnesium; phosphorus; phosphorous; iodine; fluoride; iron; manganese; selenium; chromium; molybdenum; boron; zinc; |
| *Measures* | Serum; level*; blood*; plasma; circulating; concentration*; |
